# Applying empathic communication skills in clinical practice: Medical students’ experiences

**DOI:** 10.4102/safp.v63i1.5244

**Published:** 2021-02-09

**Authors:** Elize Archer, Ilse S. Meyer

**Affiliations:** 1 Centre for Health Professions Education, Faculty of Medicine and Health Sciences, Stellenbosch University, Cape Town, South Africa

**Keywords:** empathy, undergraduate medical curriculum, educational interventions, qualitative research

## Abstract

**Background:**

Studies have demonstrated that empathic communication improves patient outcomes and helps doctors to deliver accurate symptom reports and diagnoses. These benefits emphasise the need for medical students to apply empathic communication skills during their interactions with patients. Focussed empathic communication skill workshops were introduced into the undergraduate medical students’ training at the Faculty of Medicine and Health Sciences, Stellenbosch University, South Africa. This study aimed to explore students’ perceptions of applying these empathic communication skills during their clinical practice. We were interested in determining the factors that might influence the development of empathic communication skills. The findings could help curriculum developers to optimise these workshops for inclusion in a formal medical curriculum.

**Methods:**

This study followed a qualitative, descriptive enquiry, exploring the perceptions of medical students through focus-group discussions. The students (*N* = 18) were selected using convenience sampling techniques. Recordings were transcribed, and the data were thematically analysed.

**Results:**

The two main themes identified relate to the students and the clinical learning environment. The students valued the knowledge and skills they acquired. However, feelings of emotional vulnerability, a lack of language proficiency and inadequate role modelling were highlighted as challenges when applying empathic communication during clinical practice.

**Conclusion:**

The students reported positively on the workshops as these improved both their patient and personal interactions. However, for students to develop these skills further for clinical practice, they need more intentional and supervised opportunities to practise, reflect and receive constructive feedback. These learning opportunities could help medical schools deliver graduates who can competently communicate with their patients in an empathic manner.

## Introduction

Empathic communication is widely accepted as an essential component of successful doctor–patient relationships. Medical doctors who are competent in empathic communication skills can articulate their understanding of what patients say and how they feel, and communicate this understanding to their patients.^1^ Studies have shown that this type of communication benefits patients by improving their physical and psychosocial outcomes.^2^ Clinical empathic communication involves both affective and cognitive facets.^3^ The affective facet refers to feelings and sensations in response to another person’s emotions, whereas the cognitive facet refers to identifying and communicating the emotions of others.^3^ The latter is believed to include teachable skills.^4,5^

The teaching of empathy forms part of many medical curricula globally, but the ways in which it is presented differ according to the context, resources and other factors.^4^ Whilst several educational interventions related to communication skills form part of the 6-year medical curriculum at the Faculty of Medicine and Health Sciences, Stellenbosch University, South Africa, we have recently added additional interventions that focus specifically on clinical empathy. Throughout their third year, students are exposed to two intervention sessions. During the first contact session, they attend an interactive lecture that covers various theoretical concepts relating to empathy (Box 1). Before the second, more practical contact session, conducted in the Simulation Unit, students are expected to view online study material related to empathic communication. During the 2-h session (Box 1), small groups of students are guided through various experiential learning ‘stations’, for example, mindfulness, recognising body language, an exercise with simulated patients and a structured reflective session, where students have an opportunity to share their experiences and ideas. By this time, in their third year, students are already engaged in clinical placements; therefore, learning opportunities are available to practise and develop these communication skills with patients.

BOX 1 Educational interventions for the third-year students.

- First contact session: Interactive lecture of 20 min (content discussed includes empathy versus sympathy, the relationship between patient-centredness and empathy, benefits to the patient and medical doctor and the neuroscience of empathy).
- Second contact session: Small-group experiential learning session of 2 h (students practise with simulated patients and content discussed includes active listening skills, body language, perspective-taking, mindfulness and self-care).

The same cohort of students from the third year attend a 3-h workshop on empathic communication skills (Box 2) in groups of 50–60 students during their fourth year of study. This workshop provides students with opportunities to revise and reflect on the issues covered in the third-year sessions and to discuss other challenges that might arise during communication between doctors and patients.

BOX 2 Workshop details of the fourth-year medical students.

- The first hour of the workshop provides students with an opportunity to revise the key concepts that were discussed during their third year, such as the benefits of patient-centredness, empathic communication skills and how to respond in an empathic manner.
- The second hour allows students to share their personal experiences regarding the barriers and enablers they had encountered in the clinical areas.
- In the final hour, there is a discussion on how to communicate when clinicians and patients cannot understand one another because of language barriers. To stimulate discussion, we show students a video in which a layperson acts as an interpreter for a doctor and patient who speak different languages.

A recent systematic review by Fragkos and Crampton^5^ demonstrated that the teaching of empathy amongst medical students seems to be effective; however, none of the studies cited in the review were from South Africa. Therefore, we believed that it was essential to explore this topic in the local context where large classes (*n* = 300), limited resources relating to staff, funds and time may influence the effectiveness of these interventions. This study aimed to explore students’ perceptions of applying and developing empathic communication skills during clinical practice. The findings could highlight factors that influence the development of empathic communication skills during clinical practice. Furthermore, these findings could help curriculum developers to optimise the workshops for inclusion in a formal medical curriculum.

## Methods

This descriptive study generated qualitative data that describe undergraduate medical students’ perceptions regarding the application and development of empathic communication skills during clinical practice. We used a convenience sampling approach, and students were invited to participate via email. Eighteen students (*N* = 18) were recruited and provided informed consent to participate in focus groups (FGs). Focus group 1 consisted of eight students (*n* = 8) of whom four were males (*n* = 4) and four females (*n* = 4), and FG 2 (*n* = 10) included five males (*n* = 5) and five females (*n* = 5). The duration of each FG discussion was approximately 1 h during which the students were asked to respond to prompts, such as, ‘*To what extent did the learning experiences assist you in applying and developing empathic communication skills during clinical practice?*’ and ‘*What factors influenced the application and development of empathic communication skills during clinical practice?*’ A research member (author I.S.M.), who was not involved with the students during the educational sessions, conducted the interviews, which were recorded after consent was received from all participants. The anonymised recordings were transcribed verbatim by an independent person. After reading the transcripts numerous times and guided by the research question, author I.M. coded the transcripts and identified themes,^6^ which were then reviewed and verified by author E.A.

## Findings

We identified two main themes from the data. The first theme focuses on the factors relating to the students’ personal feelings (Box 3), and the second theme concerns factors that relate to the clinical learning environment (Box 4).

BOX 3 Themes with illustrating quotes.

**Table:** 

Theme 1: Factors related to the students
**Acknowledging the positive effects of applying empathic communication skills** ‘When you enter in an empathic manner, in an open manner, the patient ends up complying with you, it can result in better interactions with patients.’ (Focus group 2; male; participant 15) ‘Not only did it enable me to interact with my patients in a meaningful way, but also my friendships in general. Not only is empathy important for us as healthcare workers in the practice itself, but just as a human life skill in general.’ (Focus group 2; male; participant 16)
**Appreciation of the teaching and learning opportunities** ‘If I wasn’t taught about empathy, I wouldn’t have known the things I know now. There are some people who may not be as privileged, to have been taught this … Now that I’ve been taught that empathy is something that you can learn. Learning something doesn’t mean you only do it once and then you’ve mastered it. It’s a continuous process.’ (Focus group 2; male; participant 15) ‘What these workshops have done is, they’ve built that foundation and shown us what we’re doing wrong or what we must focus on.’ (Focus group 1; female; participant 3) ‘… learning about empathy, you can obviously start doing it more consciously in hospital and as you become more conscious of it you realise when you’re doing it and when you’re not doing it … it really has enhanced my experience with patients …’ (Focus group 2; female; participant 11)
**Awareness of their own vulnerability** ‘Whenever you decide to be empathetic … at the same time you may subconsciously also make yourself vulnerable … I’m kind of concerned about how we would be affected.’ (Focus group 2; male; participant 15) ‘Many medical students struggle with humility…you need to become humble … that’s more of a lifestyle change that needs to happen.’ (Focus group 1; male; participant 6) ‘I understand empathy …, but how do I stop myself from taking the burdens with me home?’ (Focus group 2; female; participant 11) ‘… there’s a fine margin between showing empathy and when you become a people’s pleaser … you need to know where your boundaries lie … otherwise, emotionally, it is going to become a wreck for you as well …’ (Focus Group 2; male; participant 17)

Note: Focus group, gender (male or female), participant number.

BOX 4 Themes with illustrating quotes.

**Table:** 

Theme 2: Factors related to the clinical learning environment
**Behaviour of the clinicians** ‘The more empathic doctors that I observed, have always been good at picking up when their patients are uncomfortable, when they [medical doctors] address this, made whatever advice they gave, consequently so much more effective and impactful.’ (Focus group 1; female; participant 2) ‘… the doctor wouldn’t even give a patient 10 seconds to talk, he would just make a diagnosis and prescribe medicine and let the patient go. I genuinely felt uncomfortable, because I felt the needs of this patient weren’t addressed. So, I felt l wanted to challenge the doctor, but then at the same time, because of hierarchy and maybe being scared of the consequences, I rather just ended up moving to another consulting room and seeing a patient in my own time while he was doing his own things. That wasn’t really a nice thing for me to see.’ (Focus group 2; male; participant 14)
**Language and cultural barriers between patients and students** ‘The times I’ve struggled the most with empathy is with language difficulties … not being able to convey concerns so that the person can understand me … it does affect patient care …’ (Focus group 1; female; participant 4) ‘… in South Africa intercultural barriers, it’s major … It’s almost impossible to learn … different cultures we’re dealing with and the approach is different to each culture … that’s a major barrier … not even talking about our native South African cultures; we are talking about cultures from other parts of Africa as well.’ (Focus group 1; male; participant 7)
**Physical and time constraints** ‘… the clinical environment makes it difficult for you to find a private spot to have a proper empathic conversation with a patient, because there are 50 patients and there isn’t a bed or there isn’t even a chair for the patient to sit on … then it makes it so difficult because I struggle to find a good spot to sit down and have a proper consultation with the patient. It is not always the ideal situation … but going to the trouble to make sure the patient is as comfortable.’ (Focus group 2; female; participant 10)
‘Often doctors don’t appear to be empathic in a clinical setting, but I do think that often links to the conception that being empathic not only takes time, but also mental energy sometimes … you are so tired that at that point it seems almost too much to give more of yourself than you can at that moment.’ (Focus group 2; female; participant 11)

Note: Focus group; Gender (male or female); participant number.

### Theme 1: Factors related to the students

Three sub-themes developed from the first theme, namely, positive effects of applying empathic communication skills, appreciation of the teaching and learning opportunities and awareness of their vulnerability (Box 3).

#### Positive effects of applying empathic communication skills

Some students indicated that they had made conscious efforts to integrate empathic communication skills into their clinical practice. They realised how their efforts enhanced the quality of their relationships with patients. Students also shared that their everyday relationships with friends and family improved, and acknowledged that empathic communication was a skill that could have benefits beyond clinical practice.

#### Appreciation of the teaching and learning opportunities

Students realised that the knowledge and skills they acquired during the educational sessions helped them to communicate in an empathic manner. They understood that empathic communication was a skill that they could learn. However, they realised that this skill required considerable practice.

#### Awareness of their vulnerability

Emotional vulnerability was described by students as the uncertainty to deal with their own emotions whilst attending to patients’ needs. Some students expressed fear of not knowing how these feelings might influence their response to patients. One participant suggested that as medical students, they needed to be humble to show empathy towards their patients.

### Theme 2: Factors related to the clinical learning environment

Three sub-themes developed from the second theme, namely, the behaviour of the clinicians, language, cultural barriers between patients and students and physical and time constraints (Box 4).

#### Behaviour of the clinicians

During their clinical rotations, students appreciated the opportunity to observe the doctors interacting with their patients. Doctors who displayed humanistic interaction with their patients and, contrastingly, those who did not listen to their patients made a powerful impression on the students.

#### Language and cultural barriers between patients and students

Students realised that their care for patients could be undermined in situations where there were language or cultural barriers. Students expressed frustration about being faced with such situations.

#### Physical and time constraints

Students mentioned that there were environmental factors that made it challenging to practise empathic communication skills. These challenges included inadequate time and the shortage of private areas where one could interact confidentially with patients.

## Discussion

In this study, we explored perceptions of undergraduate medical students when attempting to apply and develop empathic communication skills during their clinical practice. We identified two key themes relating to the students and the clinical environment, which provided insights into the factors that influenced empathic communication in this setting.

### Factors related to the students

Students expressed that the workshops taught them skills that enhanced the quality of their relationships in the clinical setting and their personal lives. The students described these positive experiences as building foundational knowledge, receiving valuable feedback, meaningfully interacting with patients, enhancing their social skills, acknowledging the life-long process of learning and consciously applying the skill. These findings resonate with the study conducted by Fragkos and Crampton,^5^ which showed that empathy teaching can have positive outcomes.

Students reported some challenges when attempting to apply empathic communication in the clinical setting. They highlighted a sense of vulnerability and how they struggled to find a balance between being empathic and emotionally detached from patients. Medical students could learn how to accept this vulnerability by acknowledging that these feelings are part of the process of becoming a doctor.^7^ Students need to be encouraged to focus intentionally on empathic communication skills during their clinical practice. Of equal importance is supervised support through discussions, reflection and constructive feedback opportunities during clinical practice.^8^ This might build students’ confidence in managing the feeling of vulnerability when applying this important skill.^7^

### Factors related to the clinical learning environment

An influencing factor relating to the clinical environment became apparent when students observed clinicians’ behaviour and how this impacted the doctor-patient relationship. Some clinicians demonstrated awareness of patients’ body language and addressed patients’ needs. Students experienced this behaviour positively. Medical doctors can improve their support and management of patients by detecting problems earlier and, therefore, demonstrate empathic communication skills.^9^

In contrast, students also observed negative role modelling by some clinicians. Students observed that some clinicians spent limited time with patients and appeared to lose mental energy. Students recognised the negative effects this could have for patient-centredness, which caused the students to feel uncomfortable. Wilcox et al.^10^ specifically mentioned that certain aspects of the learning environment may impact medical students’ beliefs and behaviours and that clinicians need to explicitly model certain attitudes and behaviours to their students.

Some students were concerned about their lack of language proficiency when interacting with patients. Miscommunication was identified as a barrier to optimal patient care. According to Tongue et al.,^11^ effective communication is essential to ensure quality medical care for all patients, irrespective of culture and language differences.

Furthermore, a lack of resources in the clinical environment negatively influenced students’ application and development of empathic communication skills. These included limited space for private conversations with patients, too many patients and a shortage of available beds.

In order to determine the extent to which students had managed to develop empathic communication skills during their clinical practice, we found it valuable to refer to the integration process and transfer framework as set out by Baartman and De Bruijn.^12^ This framework describes three types of integration: the first stage, namely, ‘low-road integration’ was described by the students as the way they demonstrated their prerequisite foundational knowledge that they acquired during the teaching sessions. The second stage of integration, ‘high-road integration’, was evident when students indicated that they combined reflection with consciously integrating empathic communication skills, especially when they observed doctors interacting with their patients. Whilst students were frustrated with discrepancies as far as role modelling was concerned, both positive and negative role modelling enabled them to reflect on their actions and those of others.

For students to consciously integrate empathic communication skills, the third level of integration, namely ‘transformative integration’,^12^ is necessary. For students to reach transformative integration, they must be willing to change their approach. Students need opportunities to reflect on their existing uncertainties. Transformative integration also involves the ability to withstand social pressure. The data from this study provided no evidence that students had reached the level of transformative integration. The disabling factors mentioned by the students, such as the clinical learning environment that was not necessarily conducive to empathic communication, could have negatively impacted their reflection opportunities. These findings are similar to those of a previous study, which suggested that students need support when reflecting on their actions, behaviours and experiences with patients.^13^

## Strengths and limitations

Our study emphasises the benefits of empathy teaching and highlights factors affecting its application in the South African clinical context. As we were aware of the reciprocal experiences and assumptions as researchers, we were mindful of our own biases when analysing the data. The researchers involved the process of reflexivity to address this challenge of bias whilst collecting and analysing the data.^14^ This was ensured by being transparent regarding the steps in which the knowledge was created as well as being constantly aware of the position of the researcher in relation to the data. Furthermore, this was a small study involving participants from a single institution; however, by explaining the research design, data collection and analysis in detail, this study might enable other researchers to transfer the findings to other similar contexts.^14^ A follow-up, multi-institutional study is planned for the future.

## Conclusion

This study demonstrated evidence regarding the benefits of empathy teaching from the perspective of the student. The findings justified the continued investment of time and resources in empathic communication skills training in this context. However, in order to address the challenges faced in the clinical learning environment, provision should be made for student support through additional discussions, reflection and constructive feedback opportunities. Furthermore, we suggest that the use of interpreters may enhance patient care by facilitating communication in the multi-lingual and diverse context.
